# Awareness and Knowledge of Lead Poisoning: A Street Survey in Three Municipalities of Georgia

**DOI:** 10.3390/ijerph21121665

**Published:** 2024-12-13

**Authors:** Dali Kekelidze, Mari Malazonia, David Tsereteli, Iagor Kalandadze

**Affiliations:** 1 Ilia State University, Tbilisi 0162, Georgia; 2 National Nutrition Centre, Tbilisi 0194, Georgia; 3 David Tvilidiani Medical University, Tbilis 0159, Georgia; 4 National Center for Disease Control and Public Health, Tbilisi 0198, Georgia

**Keywords:** lead poisoning, public health, environmental exposure, Georgia

## Abstract

Lead poisoning is a serious public health problem, especially for children. Despite screening programs to reduce lead exposure, there is still a lack of knowledge about its harmful impact. The study aimed to analyze how aware people in Georgia are about lead poisoning and its health effects. In this street survey, 384 adults from three municipalities, Batumi, Ozurgeti, and Zugdidi, were interviewed from March to June 2024. We used descriptive statistics and Pearson’s chi-square test for data analysis. The majority of respondents noted that children are at high risk of lead poisoning. In all selected municipalities, people know that lead can be found in toys, but they do not know it can be found in jewelry and cosmetics, especially in Ozurgeti. Many respondents are not aware that lead poisoning can cause cardiovascular disease, anemia, and kidney failure. More educational campaigns are needed to highlight lead poisoning sources and their effects on health to protect the population.

## 1. Introduction

Lead is a chemical element marked by the Pb symbol (from the Latin plumbum), and its atomic number is 82 [1]. Pb is presented in the fourteenth group of the main group of metals in the periodic system of chemical elements. It is a naturally toxic metal found in the Earth’s crust. The primary sources of ecological pollution are the extraction of lead, industrial use, and the recycling of secondary raw materials. In some countries, lead substitute paints, dope fuel, and three-quarters of all production go into the production of lead replacement batteries. Lead is also used in many other products, such as paints, glass fittings, dishes, crystals, ceramics, jewelry, cosmetic products, and toys. Lead can enter drinking water when plumbing materials that contain lead corrode, especially where the water has high acidity or low mineral content, corroding pipes and fixtures [2]. Lead exposure can affect nearly every part of children’s bodies: (1) Children swallow lead dust from the environment by putting their fingers in their mouths and chewing on paint [3]; (2) lead is absorbed faster in children than in adults [4]; (3) children’s nervous systems are less protected from the effects of lead [5] because a child’s nervous system is still developing, making the impact of lead more toxic than on a mature brain. Exposure in early childhood can slow mental development and cause lower intelligence later in childhood [6]. Children absorb lead approximately 50% faster than adults, who absorb only about 6% [4,7] because their bodies require more nutrients like calcium and iron for growth. As lead uses the same absorption as these nutrients, their higher demand inadvertently increases lead absorption; therefore, special attention is paid to lead poisoning in children. Additionally, adults excrete 99% of lead from the body, whereas children excrete only 32% [4].

### 1.1. Lead Poisoning History

Lead air emissions have historically been predominantly from vehicle and industrial exhaust gases with varying lead levels in gasoline and diesel fuels [8]. Homes near busy roadways or fences with chipped or peeling paint sometimes have high amounts of lead in the soil [9]. It is estimated that children can be exposed to lead in soil by touching, breathing, or playing in lead-contaminated soil.

Lead is one of the metals that humans discovered. It was extracted by old Jews and Egyptians in 4000 BC, yet it was used as cargo in fishing nets, covering dishes, and cosmetic products [10,11]. Hippocrates, the father of medicine (BC 370), was the first person to associate lead with clinical symptoms [12]. From the beginning of the 20th century, lead was used widely in paints because it provided brightness, prevented mildew, and prevented corrosion [13,14]. 

In 1922, the motor car industry began using lead in petrol to increase manufacturing and protect engines from detonation [15]. In 1800, scientists described the harmful effects of lead on children and adults, linking it to lead substitute paints, petrol, and dust from polluted ground [16]. Firstly, Austrian scientist Jefferis Turner presented scientific research in 1897 which referred to the poisoning of children with lead [17]. This was followed by various studies, as a result of which, in most of Europe and Australia, from 1909 to 1930, the use of lead in paints was prohibited [10]. In the USA, from 1971, laws were made to decrease the use of lead [18].

In the USA, ethylated petrol was prohibited from 1972 to 1986 [19,20,21]. From 1978 to 1991, lead content in human blood decreased by 78%, which is linked to the reduction of lead substitutes in ethylated paint and petrol.

### 1.2. Lead Exposure as a Public Health Problem in Georgia

According to the World Health Organization (WHO), lead is one of the 10 chemical substances that cause major public health issues and require the protection of children, women, and workers from member states [22].

The Multi-Indicator Cluster Survey (MICS) was conducted in Georgia in 2018, the world’s largest household research project. It was found that 41 percent of children have blood lead levels greater than or equal to 5 micrograms per deciliter (µg/dL), and among them, 16 percent of children have blood lead levels greater than or equal to 10 micrograms per deciliter (µg/dL). Additionally, 25 percent of children have blood lead levels between 5 and 10 μg/dL [22].

According to data from the National Agency for Food, 2.38% of food samples taken from 2012 to 2017 were contaminated with lead [23]. In July 2019, under the initiative of the National Center for Disease Control and Public Health, the Center for Disease Control and Prevention of the USA, and Pure Earth, research was conducted to study the concentration of lead in various environments (soil, dust, paint, water, spices). Due to the falsification of spices, an increased lead content was revealed in spices, especially in Adjara [24].

### 1.3. Prevention Programs and Awareness

WHO works on guidelines for the prevention and management of lead content to protect children’s and adults’ health by politicians and specialists of public health. In 2002, governments appealed to remove paints containing lead entirely at the World Summit on Sustainable Development. By eradicating paints containing lead, countries will promote the achievement of the following sustainable development goal: 3.9—By 2030, substantially reduce the number of deaths and illnesses from hazardous chemicals and air, water, and soil pollution and contamination [25]. The Global Alliance to Eliminate Lead Paint (Lead Paint Alliance) was formed in 2011 to promote the production of lead substitute paints and stop their realization [26]. According to the National Center for Disease Control and Public Health of Georgia (NCDC), 1999, Georgian law mandated a maximum lead content in gasoline of 13 mg/liter. A scheduled phase-out of lead in gasoline was ordered in 2000, defining a maximum allowable lead concentration of 5 mg/L by 2005, which was later delayed until 2007 due to difficulties with enforcement and possible negative social factors. On 19 April 2019, the Georgian Government adopted Decree #869 on Early Detection and Management Measures for Toxic Effects of Lead in Children [27]. With this normative act, the relevant public agencies were instructed to take measures to reduce the toxic effects of lead in the country. As of 16 January 2024, 48% of countries have confirmed they have legally binding controls on the production, import, sale, and use of lead paints [28], and Georgia is one of them. Every October in Georgia, International Lead Poisoning Prevention Week is held to raise awareness about preventing lead poisoning. Conducting population-based studies on knowledge and attitudes regarding the harmful effects of lead poisoning is crucial to minimizing lead exposure through effective regulations and public awareness campaigns.

## 2. Material and Methods

### 2.1. Description of the Study Area

**Figure 1:**
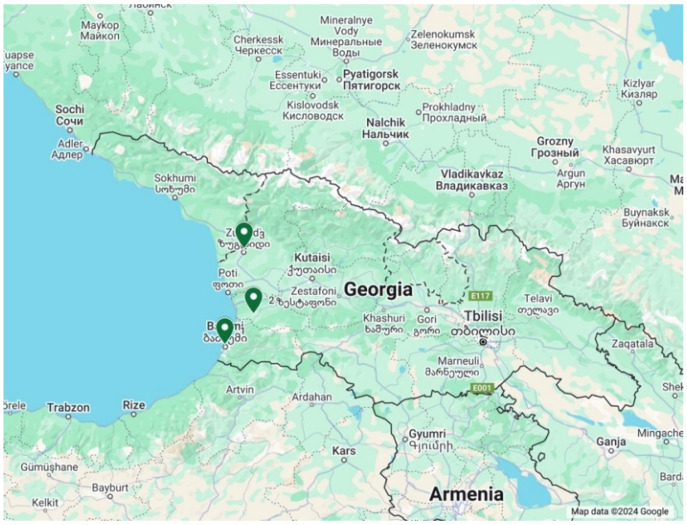
Selected study areas: 1. Batumi, 2. Ozurgeti, 3. Zugdidi.

Georgia is located at the eastern end of the Black Sea on the southern flanks of the main crest of the Greater Caucasus Mountains [29]. The geography area is 69,700 sq·km, and the total population is 4,900,961. The national language is Georgian. The most common ethnic groups are Georgian 86.8%, Azeri 6.3%, Armenian 4.5%, and other 2.3% [30]; 48% of the population are male, and 52% are female [31]. The main economic activities include cultivating agricultural products like grapes, citrus fruits, and hazelnuts; mining manganese, copper, and gold; and producing alcoholic and non-alcoholic beverages. Essentially, Georgia leads exports in copper ore, cars, fertilizers, iron alloys, and wine. It also imports cars, refined petroleum, natural gas, packaged medicine, and copper ore. The real GDP per capita is USD 22,200 (2023est) [30]. In April 2019, the Government released data on lead prevalence in children’s blood collected during the MICS. The results showed that regions in western Georgia had a much higher lead prevalence than the country’s eastern regions [32]. Therefore, we decided to conduct our study in the following western Georgian Regions: Batumi municipality, the capital of Georgia’s Autonomous Republic of Adjara [33]; Ozurgeti municipality, the capital of the west Georgian province of Guria [34]; and Zugdidi, a municipality in the west of Georgian historical province of Samegrelo [35] (Figure 1).

### 2.2. Sample Size Calculation

The sample size for the selected citizens’ interviewing population was calculated using Cochran’s sample size formula: n = z2 × P × (1 − p)/E2. where 95% of confidence level z = 1.96; the margin of error is 5% (E = 0.05), and the assumed response distribution is 50% (P = 0.5); n = (1.96)^2^ × 0.5 × 0.5/(0.05)^2^ = 3.8416 × 0.25/0.0025 = 384.16. We found that for the street survey, the sample size was approximately 384. For sufficient results, we calculated the population of each selected municipality using Geostat data for the year 2023 [36], and the sample size for each municipality was calculated in proportion to its population compared to the municipality’s total population. The final sample size per municipality by interviewing the population was Batumi—206, Ozurgeti—66, and Zugdidi—112.

### 2.3. Data Collection

For the data collection, we used questionnaires, which provided information through face-to-face interviews. The first section required sociodemographic information, the second concerned awareness of lead poisoning issues, and the third concerned knowledge of lead’s impact on health. A street survey was conducted from March to June 2024 among only adult (≥18 years) Batumi, Ozurgeti, and Zugdidi residents. We selected busy locations in each municipality, such as malls, markets, parks, and shopping streets.

### 2.4. Statistical Analysis

The interviewers’ responses were identified, imported to Excel, and analyzed using IBM SPSS version 23 (IBM, Armonk, NY, USA). Descriptive statistics were used to summarize the study data findings. Pearson’s chi-square test was used to determine significant associations between different variables.

## 3. Results and Discussion

A total of 384 people participated in the study. The mean age was 39.2 years, with a standard deviation of 14.5 years, indicating a moderate level of variability in the ages; most participants were female, 53.4%. Participants were distributed by the following municipalities: Batumi (53.6%), Zugdidi (29.2%), and Ozurgeti (17.2%); 63.8% of the participants had children. Nearly 51.0% of the participants had secondary education, and 63.3% were employed; 62.8% of the participants did not have children under 6 living at home. Approximately 52.4% of participants refused that their family’s monthly income is above GEL 1500. The characteristics of interviewing respondents are presented in Table 1.

**Figure 2:**
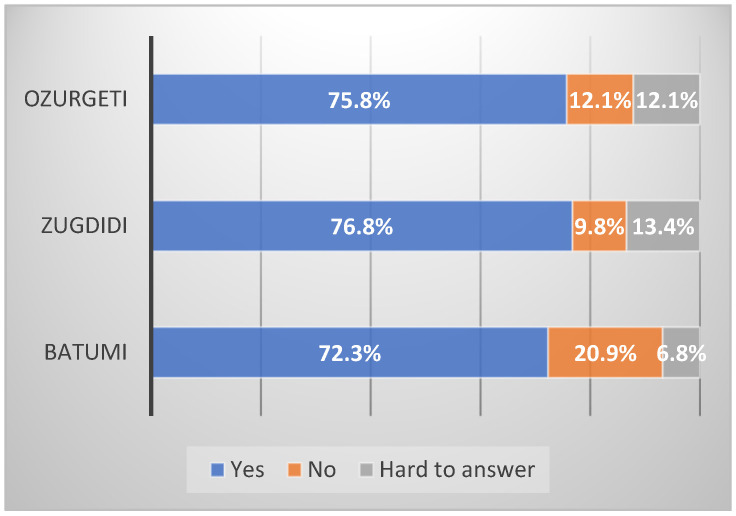
Do you believe that children are at a higher risk of lead poisoning?

Figure 2 shows participants’ opinions from three municipalities about whether or not children are at a high risk of lead poisoning. Most participants think children are at a high risk of lead poisoning. It means that general participants are aware that children are at a higher risk of lead poisoning. This was in line with a study conducted in Saudi Arabia, which shows that 50.5% of the participants think that children are most at risk for lead poisoning [37].

**Figure 3:**
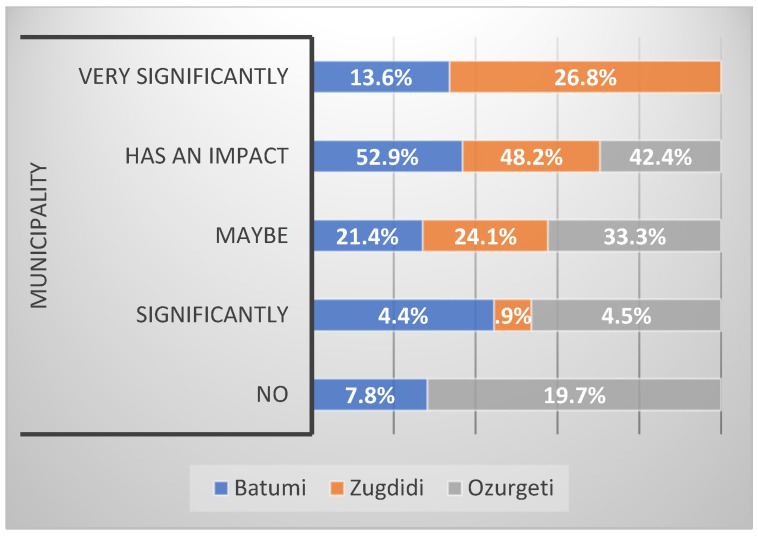
Do you think lead affects health as a metal?

Figure 3 illustrates what participants from three municipalities think about whether or not lead affects health. Most participants from Batumi (52.9%) think lead is a metal that impacts health. In Zugdidi, 48.2% think lead has an impact, while 26.8% think it affects health significantly. In Ozurgeti, none of the respondents answered that lead affects health significantly, but 42.4% think it has an impact. Also, in a study in South Western Nigeria, most discussants stated that they believe lead is poisonous and dangerous to health [38].

Lead poisoning can have a harmful impact on a child’s development and behavior. Children with greater lead levels may also have problems with learning and reading, delayed growth, and hearing loss [39]. Lead may be used in plastic toys to stabilize molecules from heat [40]. Exposure to lead is associated with multiple sources such as petrol, industrial processes, paint, solder in canned foods, water pipes, and pathways: air, household dust, street dirt, soil, water, and food. It is also found in jewelry and decorative items [41]. Applying lead-containing cosmetics several times a day or daily can potentially add to significant exposure levels [42]. Thus, Table 2 describes interviewing respondents’ awareness of lead poisoning issues by municipality. In Batumi, 18%, Zugdidi, 12.5%, and Ozurgeti, 18.2% of respondents think learning disabilities are major symptoms associated with lead poisoning. Growth and development problems are mostly recognized symptoms in Batumi (54.9%) and in Zugdidi (42.9%) and less recognized in Ozurgeti (31.8%). In all municipalities, fewer people mentioned that speech difficulties, low academic performance at school, and difficulty in reading are major symptoms associated with lead poisoning. More respondents in Batumi (78.6%) and Zugdidi (70.5%) said they had received information about lead poisoning, compared to 57.6% in Ozurgeti. These results suggest that in Ozurgeti, more awareness companies are needed. Across the municipalities, most respondents in Batumi (85.9%), Zugdidi (79.5%), and Ozurgeti (77.3%) think lead-substitute paints are related to lead poisoning. A majority of respondents in all municipalities are less informed that lead poisoning is linked to lead-contaminated air and that lead poisoning is associated with lead-contaminated soil. Respondents in Batumi (50.5%) believe that lead-substitute foods are linked to lead poisoning, and only Zugdidi (24.1%) and Ozurgeti (18.2%) show a lower percentage. In Batumi (48.5%), Zugdidi (18.8%), and Ozurgeti (21.2%), respondents believe that household utensils containing lead can be linked to lead poisoning.

In every municipality interviewed, respondents are aware that lead-substitution toys are related to lead poisoning. However, all municipality respondents are not aware that lead poisoning is associated with lead replacement jewelry and are also not mindful that lead-containing cosmetic products are linked to lead poisoning.

Table 3 shows respondents’ knowledge of the health effects of lead poisoning. For cardiovascular disease, interviewing respondents do not have the knowledge that lead poisoning can cause it. The respondents who said NO are as follows: In Batumi, 46.1%; Zugdidi, 42.9%; and in Ozurgeti, 19.7%. Most respondents answered that they do not know if lead poisoning can cause cardiovascular disease. Knowledge of lead poisoning causing anemia was poor. The higher percentage of those who answered “NO” was from Batumi (35.0%). The most common answer by respondents in all municipalities was “Don’t Know.” The distribution is as follows: Batumi (48.5%), Zugdidi (38.4%), and Ozurgeti (68.2%). In the case of whether lead poisoning can cause kidney failure, most respondents are not aware. The highest percentage of those who said “NO” was observed in Zugdidi (57.1%) and Batumi (47.6%). The majority of interviewing respondents in all municipalities answered “Don’t know” (Batumi (49.5%), Zugdidi (41.7%) and Ozurgeti (69.7%)).

## 4. Conclusions

The results show that most respondents believe that children are at high risk of lead poisoning and that lead affects health as a metal. Awareness regarding lead poisoning sources, such as contaminated air, soil, and household utensils, is higher in Batumi compared to Zugdidi and Ozurgeti. Major respondents by municipalities were informed that lead-containing toys could be a source of lead poisoning. However, they were not informed about the risks of lead poisoning from lead-containing jewelry or cosmetic products. The respondents’ awareness of health risks related to lead poisoning in all cities is notable, including cardiovascular disease, anemia, and kidney failure. Public health specialists need to launch more educational campaigns to enhance people’s understanding and awareness of the harmful effects of lead poisoning.

## 5. Limitations of the Study

Our study sample was too small, which limits the significance of the study findings. We strongly believe that our findings will push governmental and non-governmental authorities to take action and conduct larger population-based research on lead poisoning awareness and knowledge.

## Figures and Tables

**Table 1:** Characteristics of the interviewing respondents.

Characteristics	N = 384
Age	39.2 ± 14.5 ^a^
Gender	
Female	205 (53.4%)
Male	179 (46.6%)
Municipality	
Batumi	206 (53.6%)
Zugdidi	112 (29.2%)
Ozurgeti	66 (17.2%)
Parental status	
Has children	245 (6 3.8%)
Has no children	139 (36.2%)
Education	
Incomplete secondary education	1 (0.3%)
secondary education	196 (51.0%)
Bachelor’s Degree	171 (44.5%)
Student	15 (3.9%)
Employment Status	
Employed	243 (63.3%)
Unemployed	40 (10.4%)
Pensioner	4 (1.0%)
Student	43 (11.2%)
Housewife	54 (14.1%)
Child under the age of 6 living in respondent’s home	
Yes	143 (37.2%)
No	241 (62.8%)
Monthly family income categories(GEL)	
1: <250;	1 (0.3%)
2: 251–500	11 (2.9%)
3: 501–1000	44 (11.5%)
4: 1001–1500	70 (18.2%)
5: >1500	201 (52.4%)
Don’t know	28 (7.3%)
Hard to answer	29 (7.6%)

^a^ Mean ± SD.

**Table 2:** Interviewing respondent’s awareness of lead poisoning issues by municipality.

Characteristics	Batumi	Zugdidi	Ozurgeti	Pearson’s Chi-Squared Test
A major symptom associated with lead poisoning				0.000
Learning disability	18.0%	12.5%	18.2%	
Disturbance of the growth and development process	54.9%	42.9%	31.8%	
Concentration problems	9.2%	33.0%	7.6%	
Speech difficulties	4.9%	4.5%	4.5%	
Difficulty in reading	4.4%			
Hard to answer	8.7%	7.1%	37.9%	
Receiving information about lead poisoning				0.026
Yes	78.6%	70.5%	57.6%	
No	20.4%	29.5%	42.4%	
Hard to answer	1.0%			
Are lead-substitute paints related to lead poisoning?				0.109
Yes	85.9%	79.5%	77.3%	
No	6.8%	3.6%	6.1%	
Hard to answer	7.3%	17.0%	16.7%	
Whether or not lead poisoning is linked to lead-contaminated air?				0.000
Yes	40.3%	5.4%	15.2%	
No	47.1%	74.1%	62.1%	
Hard to answer	12.6%	20.5%	22.7%	
Whether or not lead poisoning is associated with lead-contaminated soil?				0.000
Yes	27.7%		1.5%	
No	60.7%	79.5%	75.8%	
Hard to answer	11.7%	20.5%	22.7%	
Whether or not lead poisoning is linked to lead-substitute foods?				0.000
Yes	50.5%	24.1%	18.2%	
No	37.9%	61.6%	56.1%	
Hard to answer	11.7%	14.3%	25.8%	
Are household utensils containing lead linked to lead poisoning?				0.000
Yes	48.5%	18.8%	21.2%	
No	39.8%	57.1%	50.0%	
Hard to answer	11.7%	24.1%	28.8%	
Are lead-substitution toys related to lead poisoning?				0.000
Yes	76.7%	54.5%	59.1%	
No	16.5%	33.9%	16.7%	
Hard to answer	6.8%	11.6%	24.2%	
Whether or not lead poisoning is associated with lead replacement jewelry?				0.000
Yes	29.6%		6.1%	
No	51.5%	74.1%	57.6%	
Hard to answer	18.9%	25.9%	36.4%	
Are lead-containing cosmetic products linked to lead poisoning?				0.000
Yes	44.7%	3.6%	3.0%	
No	40.8%	71.4%	54.5%	
Hard to answer	14.6%	25.0%	42.5%	

**Table 3:** Interviewing respondent’s knowledge of lead impact on health.

Characteristics	Batumi	Zugdidi	Ozurgeti	Pearson’s Chi-Squared Test
Do you think lead poisoning can cause cardiovascular diseases?				0.000
Yes	5.3%	17.0%	15.2%	
No	46.1%	42.9%	19.7%	
Don’t know	48.5%	40.2%	65.2%	
Do you think lead poisoning can cause anemia?				0.000
Yes	16.5%	37.5%	15.2%	
No	35.0%	24.1%	16.7%	
Don’t know	48.5%	38.4%	68.2%	
Do you think lead poisoning can cause kidney failure?				0.004
Yes	2.9%	1.8%		
No	47.6%	57.1%	30.3%	
Don’t know	49.5%	41.1%	69.7%	

## Data Availability

Data are available upon reasonable request from the corresponding author.
